# Butyrate producers, “The Sentinel of Gut”: Their intestinal significance with and beyond butyrate, and prospective use as microbial therapeutics

**DOI:** 10.3389/fmicb.2022.1103836

**Published:** 2023-01-12

**Authors:** Vineet Singh, GyuDae Lee, HyunWoo Son, Hong Koh, Eun Soo Kim, Tatsuya Unno, Jae-Ho Shin

**Affiliations:** ^1^Department of Applied Biosciences, Kyungpook National University, Daegu, Republic of Korea; ^2^Department of Pediatrics, Severance Fecal Microbiota Transplantation Center, Severance Hospital, Yonsei University College of Medicine, Seoul, Republic of Korea; ^3^Department of Internal Medicine, School of Medicine, Kyungpook National University, Daegu, Republic of Korea; ^4^Faculty of Biotechnology, School of Life Sciences, SARI, Jeju National University, Jeju, Republic of Korea; ^5^Department of Integrative Biotechnology, Kyungpook National University, Daegu, Republic of Korea

**Keywords:** butyrate producers, microbial homeostasis, gut epithelial barrier, immunomodulation, gut inflammation, colorectal cancer, gut-organ axis

## Abstract

Gut-microbial butyrate is a short-chain fatty acid (SCFA) of significant physiological importance than the other major SCFAs (acetate and propionate). Most butyrate producers belong to the Clostridium cluster of the phylum Firmicutes, such as *Faecalibacterium*, *Roseburia*, *Eubacterium*, *Anaerostipes*, *Coprococcus*, *Subdoligranulum*, and *Anaerobutyricum*. They metabolize carbohydrates *via* the butyryl-CoA: acetate CoA-transferase pathway and butyrate kinase terminal enzymes to produce most of butyrate. Although, in minor fractions, amino acids can also be utilized to generate butyrate *via* glutamate and lysine pathways. Butyrogenic microbes play a vital role in various gut-associated metabolisms. Butyrate is used by colonocytes to generate energy, stabilizes hypoxia-inducible factor to maintain the anaerobic environment in the gut, maintains gut barrier integrity by regulating Claudin-1 and synaptopodin expression, limits pro-inflammatory cytokines (IL-6, IL-12), and inhibits oncogenic pathways (Akt/ERK, Wnt, and TGF-β signaling). Colonic butyrate producers shape the gut microbial community by secreting various anti-microbial substances, such as cathelicidins, reuterin, and β-defensin-1, and maintain gut homeostasis by releasing anti-inflammatory molecules, such as IgA, vitamin B, and microbial anti-inflammatory molecules. Additionally, butyrate producers, such as *Roseburia*, produce anti-carcinogenic metabolites, such as shikimic acid and a precursor of conjugated linoleic acid. In this review, we summarized the significance of butyrate, critically examined the role and relevance of butyrate producers, and contextualized their importance as microbial therapeutics.

## Role of butyrate-producing gut-commensals

The human gut harbors an enormous number of microbes, approximately 38 × 10^12^ in total (Sender et al., 2016), comprising genetic material that is comparable to the human genome itself (Manson et al., 2008). This complex gut microbiome contains both aerobic and anaerobic commensal microbes, but anaerobic microbes constitute 99% of the gut microbiota (Nagpal et al., 2017). The gut environment is predominantly anaerobic, providing a suitable ecological niche for anaerobic commensals. The gut microbiome is host-specific, and even among healthy individuals, it varies with geographical location, race, ethnicity, and diet (Gupta et al., 2017). These host-specific gut communities interact with each other through a number of metabolites, which in turn promote gut health (Lin and Zhang, 2017; Krautkramer et al., 2021). Gut microbes also affect the overall health of the host by participating in various metabolic pathways, regulating gene expression, and synthesizing beneficial bioactive compounds, such as short-chain fatty acids (SCFAs), amines, secondary bile acids, and vitamins. In the gut, SCFAs are the major beneficial metabolites produced by gut microbes through metabolizing indigestible dietary fibers. SCFAs are fatty acids with fewer than six carbon atoms and comprise three major forms, i.e., acetate (60%), propionate (20%), and butyrate (20%) (Chambers et al., 2018). Among them, butyrate has been considered of significant importance, as it is involved in several functions of physiological importance, such as trans-epithelial transport, amelioration of mucosal inflammation, alleviation of oxidative stress, enforcement of the epithelial barrier, and protection against colorectal cancer (CRC) (Hamer et al., 2008). The microbial origin butyrate is mainly synthesized by certain anaerobic commensal microbes belonging to the Clostridium cluster (Clostridium_IV and Clostridium_XIVa) of the phylum Firmicutes (Manson et al., 2008). In addition, it is also known that certain commensals convert bacterial metabolites such as lactate and acetate into butyrate *via* the acetyl-CoA pathway (Bui et al., 2015; Belzer et al., 2017).

In the gut, colon is the primary site of fermentation of indigestible fibers by fibrolytic, butyrate-producing microbes, such as *Roseburia intestinalis*, *Faecalibacterium prasunitzi*, *and Eubacterium*, which are sensitive to the presence of oxygen (Manson et al., 2008). Colonic butyrate is actively transported to colonocytes by monocarboxylate transporters, where the majority (~70%) of transported butyrate is used to generate energy *via* the citric acid cycle. Non-metabolized butyrate, on the other hand, is transported to the hepatic portal system (Zheng et al., 2017) where butyrate acts as an energy source for hepatocytes, and from there, it is transported to peripheral tissue and systemic circulation. The concentration of butyrate in portal circulation is around 30 μM, and falls near 0.2–15 μM in the systemic circulation, which is almost 2% of the colonic butyrate concentration (Dalile et al., 2019).

The lower level of butyrate producers is continuously found to be associated with various ailments, such as *Roseburia* in colorectal cancer and inflammatory bowel disease (Sun et al., 2020; Wu et al., 2022), butyrate-producing *Coprococcus* in pregnant preeclampsia patients (Altemani et al., 2021), and *Faecalibacterium* in gut inflammation (Fujimoto et al., 2013). Therefore, the level of butyrate producers should be considered to be of therapeutic importance, which has even promoted its oral administration in various studies (Vieira et al., 2012; Chen et al., 2018; Liu et al., 2019). Additionally, butyrate producers are present in the human gut, and their proportion can be enhanced by selecting a suitable diet and healthy lifestyle, thus facilitating the maintenance of overall gut health.

## Microbial butyrate and its fate in the gut

**Figure 1:**
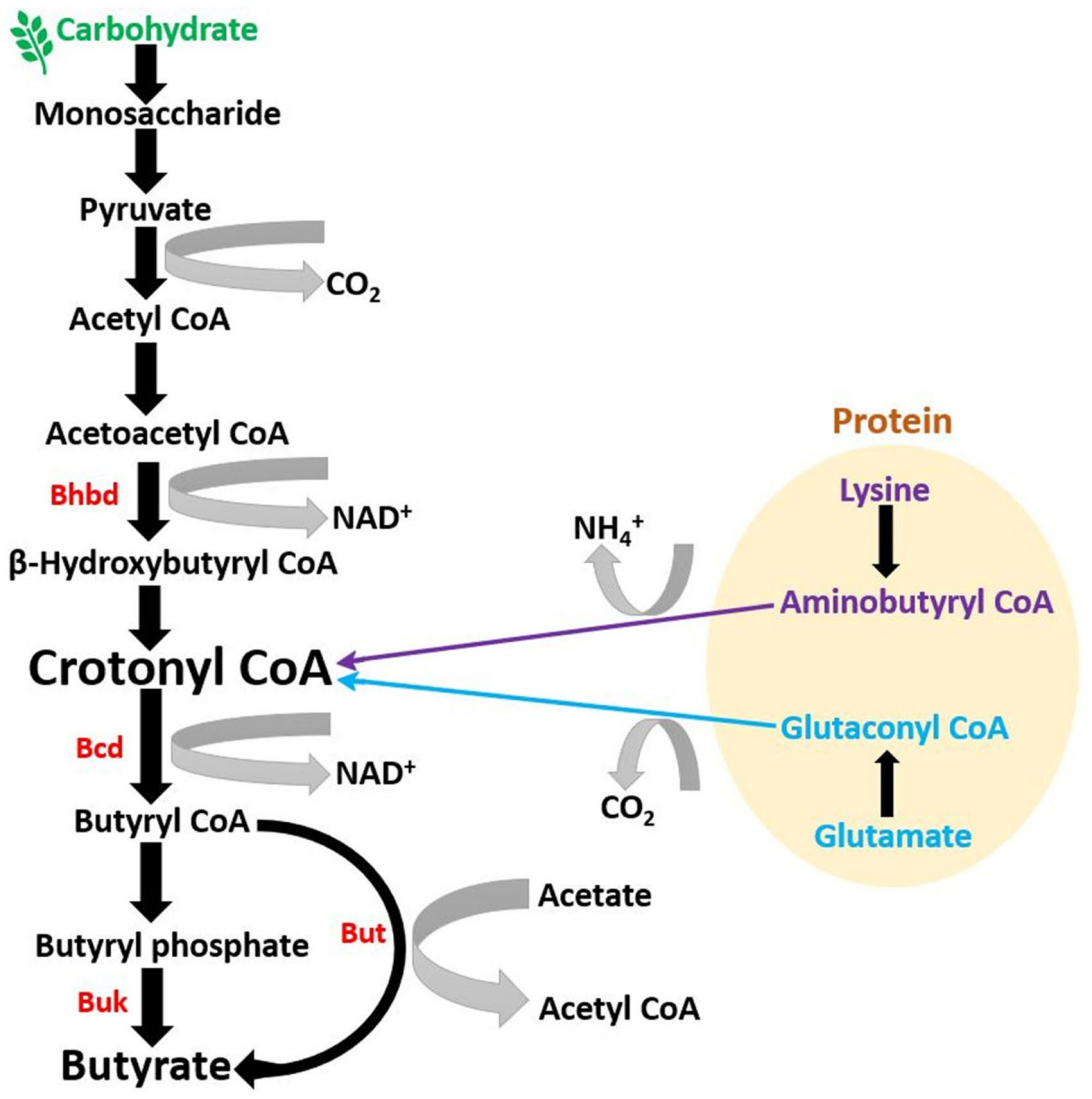
Microbial pathway to generate butyrate in gut: Majority of butyrate in the colon is generated by the metabolization of dietary fibers, primarily of carbohydrate origin (BHBD, β-hydroxybutyryl-CoA dehydrogenase; Bcd, butyryl-CoA dehydrogenase; But, butyryl-CoA: acetate CoA-transferase; Buk, butyrate kinase).

Studies suggest that initial butyrate-producing communities, i.e., initial butyrate producers in infant gut, such as Clostridiaceae, Lachnospiraceae, and Ruminococcaceae spp., might be introduced into the human gastrointestinal tract *via* resistant microbial endospores (Appert et al., 2020). A recent study on a Swiss-cohort confirmed that *Eubacterium hallii*, a member of the family Lachnospiraceae, is one of the earliest butyrate producers in the gut of infants (Schwab et al., 2017). This is also supported by a study on Swiss, Venezuela, Malawi, and USA populations, which confirmed the human milk oligosaccharide metabolizing ability of *Eubacterium Hallii* (Schwab et al., 2017). The majority of butyrate producers are gram-positive and come under Clostridium clusters IV and XIVa of the phylum Firmicutes (Manson et al., 2008; Table 1). These microbial communities comprise a significant population of butyrate-producers, including various butyrogenic species of *Eubacterium*, *Faecalibacterium*, and *Roseburia* (Manson et al., 2008; Louis and Flint, 2009). Among all butyrate producers, *Faecalibacterium prausnitzii* is most abundant in fecal samples (~ 5%) (Miquel et al., 2013), and its proportion can increase up to 13–17.6% (Manson et al., 2008). Other major butyrate producers in fecal gut microbiota are *Eubacterium rectale*, *Eubacterium Hallii*, and *Roseburia intestinalis*, which can constitute up to ~13% (Rivière et al., 2016), 2.4% (mean, 0.6%), and 0.9–5% (mean, 2.3%), respectively (Hold et al., 2003). In smaller fractions, various other butyrate producers are also present in the gut, which produce butyrate by utilizing different dietary oligosaccharides, polysaccharides, and metabolic intermediates (Table 1). Although the majority of butyrate-producing microbes belong to the phylum Firmicutes, studies have suggested that certain members of the phyla Actinobacteria, Bacteroidetes, Fusobacteria, and Proteobacteria can also produce butyrate (Vital et al., 2014). During fermentation, butyrate producers cause substrate-level phosphorylation of the dietary substrate to generate energy in the form of ATP, which results in the formation of multiple end-products, including butyrate (Louis and Flint, 2009). In the human gut, the majority of microbial butyrate is synthesized from carbohydrate metabolism *via* butyryl-CoA: acetate CoA-transferase pathway (but) and butyrate kinase (buk) pathway, of which the but-pathway is predominant (Vital et al., 2013); (but) and (buk) are derived from the genes encoding enzymes involved in the terminal steps of microbial butyrate synthesis (Altemani et al., 2021). *Radioisotope analysis of human fecal microbiota has shown that the* majority of butyrate in the gut is produced from carbohydrates through the Embden-Meyerhof-Parnas pathway (glycolysis) *via* acetyl-CoA (Miller and Wolin, 1996; Louis and Flint, 2009; Figure 1). *During this process, two molecules of acetyl-CoA combine to form a butyrate molecule* (Miller and Wolin, 1996), and the transformation of crotonyl-CoA to butyryl-CoA is the main energy generation step (Tsukuda et al., 2021; Figure 1). *In addition to carbohydrates, in minor fraction, butyrate can also be synthesized from proteins via glutamate, lysine, glutarate, and 4-aminobutyrate pathways* (Louis and Flint, 2017; Vital et al., 2017; Mallott and Amato, 2022). Furthermore, butyrate is transported into colonocytes in the gut epithelium *via* monocarboxylate transporter 1 (MCT1) (Cuff et al., 2002), where it participates in various activities, including stabilization of hypoxia-inducible factor (HIF), inhibition of histone deacetylase (HDAC), and regulation of specific G-protein coupled receptors, which will be discussed later.

**Table 1:** Major butyrate producers in the human gut and their relevance.

Butyrate producer					
Phylum	Sub-cluster	Genus	Species	Relevance	Reference
Firmicutes	Clostridium IV Or Clostridium leptum group	*Faecalibacterium*	*F. prasunitzi*	Most abundant butyrate producer	Louis and Flint (2009)
		*Subdoligranulum*	*S. variabile*	Metabolizes calprotectin	Kamp et al. (2022)
		*Anaerotruncus*	*A. colihominis*	Degrade mucin	Raimondi et al. (2021)
		*Ruminococcus*	*R. bromii*	Key fermenter of resistant starch	Ze et al. (2012)
			*R. callidus*	Degrades complex polysaccharides such as starch or xylan	Chassard et al. (2012)
			*R. champanellensis*	Most efficient cellulolytic bacterium in human colon	Chassard et al. (2012)
	Clostridium XIVa or Clostridium coccoides group	*Roseburia*	*R. intestinalis*	Major Xylan degrader in human gut	Leth et al. (2018), Mirande et al. (2010)
			*R. faecis*	Utilizes fructose, glucose, maltose, cellobiose, raffinose, xylose, sorbitol, melibiose and amylopectin starch; but not Arabinose, and sucrose	Duncan et al. (2006)
			*R. hominis*	Utilizes arabinose, fructose, glucose, maltose, cellobiose, xylose and glycerol; but not Sucrose, sorbitol, oat spelt xylan, amylopectin starch and inulin (dahlia)	Duncan et al. (2006)
			*R. inulinivorans*	Utilizes inulin (dahlia), fructose, glucose, and maltose cellobiose, and amylopectin; but not rabinose, raffinose, xylose, glycerol, sorbitol and oat spelt xylan	Duncan et al. (2006)
		*Anaerostipes*	*A. caccae*	Utilizes Lactate to produce butyrate	Duncan et al. (2004)
			*A. hadrus*	Utilizes D-Lactate (not L-Lactose) and acetate to produce butyrate	Allen-Vercoe et al. (2012)
			*A. butyraticus*	Utilizes fructooligosaccharide (FOS) to produce butyrate	Endo et al. (2022)
			*A. rhamnosivorans*	Utilizes lactate and acetate for butyrate generation	Bui et al. (2019)
		*Butyrivibrio*	*B. fibrisolvens*	Utilizes cellulose	Rodríguez Hernáez et al. (2018), Paillard et al. (2007)
		*Eubacterium*	*E. rectale*	Metabolizes sulfonated monosaccharide (sulfoquinovose) present in green vegetables; Dahlia inulin is specifically catabolized	Hanson et al. (2021)
			*E. ramulus*	Metabolizes variety of flavonoids	Schneider and Blaut (2000), Braune et al. (2001)
			*E. hallii*	Utilizes glucose and the intermediates acetate and lactate, for butyrate generation	Engels et al. (2016a)
			*E. limosum*	Transformation of 8-prenylanringenin (phyto-estrogen) from iso-xanthohumol	Possemiers et al. (2008)
		*Coprococcus*	*C. cactus*	Metabolizes fructose; cross-feed on fermentation products (acetate, lactate) to produce butyrate	Reichardt et al. (2014), Alessi et al. (2020)
			*C. eutactus*	Metabolizes β-glucan, cellobiose and lichenan	Alessi et al. (2020)
			*C. comes*	Metabolizes glucose	Alessi et al. (2020)
		*Anaerobutyricum*	*A*. *soehngenii*	Utilizes D-and L-lactate and acetate to produce butyrate	Gilijamse et al. (2020)

## Impact of butyrate producers on neighboring gut microbial communities

**Figure 2:**
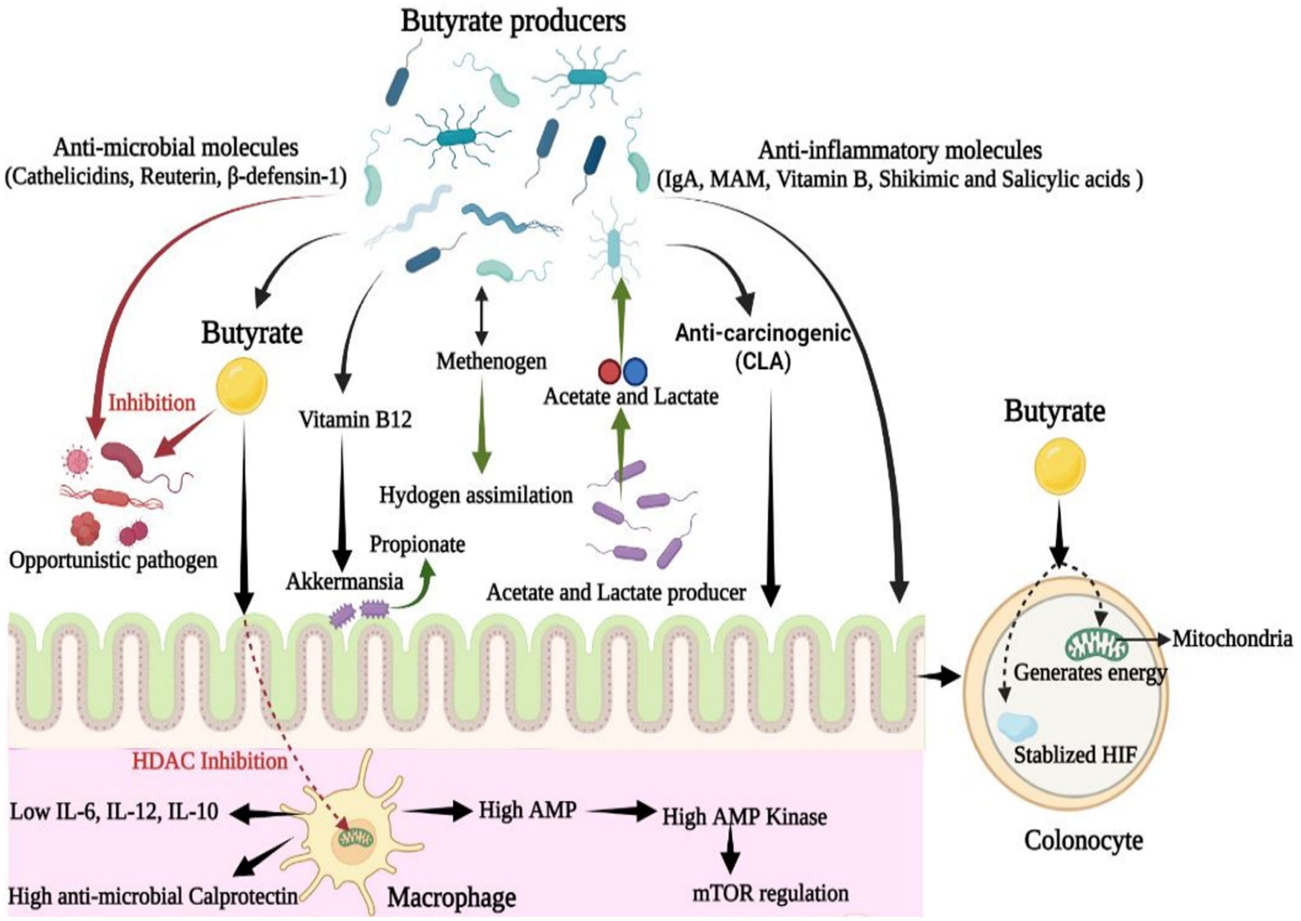
Dynamic role of butyrate producing microbial communities in gut: Along with butyrate, butyrate-producing communities also produce various bioactive molecules that are anti-microbial, anti-inflammatory, and anti-carcinogenic in nature. These molecules are of therapeutic importance in alleviating gut-associated disorders and maintaining gut-homeostasis (CLA, Conjugated Linoleic Acid; IL, Interleukin; MAM, Microbial Anti-inflammatory Molecule).

In the gut, butyrate-producing microbial communities play a crucial role in maintaining a healthy gut environment as they restrict the entry and establishment of other microbes, especially pathogenic microbes. Butyrate is used by colonocytes to generate energy which increases epithelial oxygen consumption (Litvak et al., 2018). As a result, the presence of butyrate producing bacteria helps maintain an anaerobic environment in the gut, which further prevents the colonization of opportunistic aerobic pathogens, such as *Salmonella and E. coli* (Manson et al., 2008; Parada Venegas et al., 2019). Butyrate also regulates the production of cathelicidins, a polycationic peptide that participates in mammalian innate immunity and exhibits broad-spectrum antimicrobial activity against potential gut pathogens (van Vliet et al., 2010; Kościuczuk et al., 2012; van Harten et al., 2018). Moreover, butyrate-producing bacteria such as *E. hallii* produces reuterin, a broad-spectrum antimicrobial agent with yeast inhibition activity (Engels et al., 2016b) while metabolizing glycerol to 3-hydroxypropionaldehyde (Figure 2). These anti-microbial agents limit the incursion or abundance of potential pathogens and thus, help maintain a healthy gut microbiome.

Butyrate produced in the gut shapes the gut microbial community *via* regulating IgA secretion and by limiting the hyperresponsiveness of macrophages toward colonic commensals to maintain their abundance (Chang et al., 2014; Isobe et al., 2020). Butyrate regulates colonic macrophages present in the lamina propria by inhibiting HDAC, and limits the generation of proinflammatory IL-12 and IL-6, as well as antimicrobial nitric oxide from lipopolysaccharide-stimulated macrophages (Chang et al., 2014; Kibbie et al., 2021). Butyrate enhances the GPCR-independent antimicrobial activity of macrophages *via* metabolites, as evidenced by a study that showed that macrophages grown in the presence of microbial butyrate upregulated the expression of antimicrobial protein calprotectin but showed lowered expression of anti-inflammatory IL-10 (Schulthess et al., 2019; Jukic et al., 2021; Figure 2). Additionally, microbial butyrate significantly enhances the ability of macrophages to eliminate possible pathogens, such as *Salmonella enterica* and *Citrobacter rodentium* (Flemming, 2019). Thus, butyrate bolsters gut defense against invasive pathogens without causing tissue-damaging inflammation or hyper-responsiveness. Butyrate-induced macrophages also exhibit higher levels of AMP, an inducer of AMP-kinase (AMPK), which inhibits mammalian target of rapamycin (mTOR), the master regulator protein kinase of autophagy, which is associated with cancer, insulin resistance, and other diseases (Schulthess et al., 2019; Figure 2).

*In vitro* and *in vivo* studies have also shown that butyrate producers participate in vitamin biosynthesis, especially vitamin B complex biosynthesis. For example, *Eubacterium hallii* produces vitamin B12, which is symbiotically utilized by *Akkermansia* to produce propionate (Belzer et al., 2017; Pham et al., 2021; Figure 2). The vitamin B complex acts as an essential cofactor in various metabolic activities and is also associated with the regulation of immunological homeostasis in the host (Yoshii et al., 2019). A cross-feeding relationship is also reported between butyrogenic genera, such as *Faecalibacterium*, *Roseburia*, *Anaerostipes*, *Eubacterium*, and probiotic *Bifidobacterium* (Rivière et al., 2016). For example, *Bifidobacterium* produces lactate and acetate, which are further utilized by butyrogenic microbes, such as *E. Hallii*, to generate butyrate; this in turn supports the abundance of Bifidobacterium (Louis and Flint, 2009; Schwab et al., 2017). Similarly, *Anaerostipes hadrus* and *Anaerobutyricum hallii*, members of the family Lachnospiraceae, utilize lactate and acetate to produce butyrate in the gut (Duncan et al., 2004).

## Importance of butyrate producers in maintaining the gut epithelial barrier

The intestinal epithelium is a single-layer structure covered by a mucous layer and functions as the first line of defense against gut pathogens. The cells of intestinal epithelium are interconnected with tight junctions. The intestinal epithelium contains mucous-secreting goblet cells that provide barrier protection by secreting mucus, which also functions as a reservoir of immunoglobulin IgA and antimicrobial peptides (Martens et al., 2018). The mucous layer is composed of mucin, and in colon *MUC2* is the primary mucin-producing gene (Martens et al., 2018). The mucous layer adhering to the gut epithelium is thick and limits the microbial growth near the epithelial layer, whereas the outer mucous layer is less dense and suitable for the growth of different commensals, such as *Akkermansia muciniphila*, *Faecalibacterium*, *and Eubacterium rectale* (Maier et al., 2015; Martens et al., 2018). Some harmful microbes can decrease mucus thickness by degrading it, thereby allowing pathogens to enter the gut; for example, *Vibrio cholerae* secretes hemagglutinin protease that possesses mucolytic activity. Cholera-causing bacteria also secrete zonula occludens toxin, which further hampers epithelial integrity by acting on tight junctions (Martens et al., 2018). Another microbe, *Clostridium perfringens*, disrupts tight junctions by secreting endotoxins (Saitoh et al., 2015). Additionally, decreased abundance of butyrate producers leads to compromised defense and dysfunctional gut epithelium as observed in the case of *Clostridium difficile* infection (Antharam et al., 2013).

*Faecalibacterium*, a major butyrate producer in the human gut, enhances mucus formation by increasing goblet cell differentiation and expression of genes related to mucin glycosylation (Wrzosek et al., 2013). Furthermore, clinical studies have demonstrated rapid recovery in patients with cholera after oral administration of resistant starch, a butyrate precursor (Canani et al., 2011). In addition, butyrate produced by bacteria in the gut accelerates mitochondria-dependent oxygen consumption in gut epithelial cells, which stabilizes HIF. Butyrate itself also inhibits HIF-prolyl hydroxylase that degrades HIF (Wang et al., 2021). Stabilized HIF regulates the tight junction protein claudin-1, *MUC2* expression, and generation of antimicrobial peptide beta defensin-1 (DEFB1) (Zheng et al., 2017; Wang et al., 2021). Butyrate also regulates the immunological aspect of barrier function as it tightens the intestinal epithelial cell barrier *via* inducing anti-inflammatory cytokine IL-10RA-dependent suppression of claudin-2 protein, which forms paracellular channels in tight junctions and increases gut permeability (Zheng et al., 2017; Zhu et al., 2019). A recent study also demonstrated the role of butyrate in the regulation of actin-binding protein synaptopodin (SYNPO), which is expressed in gut epithelial tight junctions and is crucial for gut-barrier integrity (Wang et al., 2020).

## Protective role of butyrate producers against bowel inflammation

Based on their severity, inflammatory diseases of the gut can be categorized into irritable bowel syndrome (IBS) and inflammatory bowel disease (IBD). IBS is characterized by cramps, bloating, diarrhea, and/or constipation (Camilleri et al., 2016). There are no biological markers to confirm it; moreover, this condition does not pose major discomfort to the patients. Normally, IBS patients are identified using a questionnaire prepared by medical staff (Werlang et al., 2019). In contrast, IBD is a generic term for more severe conditions, such as Crohn’s disease and ulcerative colitis (Franzosa et al., 2019), which cause inflammation and ulcers in the intestine, rectal bleeding, anemia, and diarrhea. Incidentally, decreased butyrate levels have often been reported in both IBS and IBD. In the case of IBD, butyrate producers play important roles as they increase mucus production from goblet cells to strengthen the intestinal mucous barrier and regulate the expression of tight junction proteins *via* butyrate to restrict the harmful penetration through the gut (Pozuelo et al., 2015; Pascal et al., 2017; Dalile et al., 2019; Schirmer et al., 2019). Similarly, in the case of IBS lower number of butyrate producers result in a reduced availability of butyrate and thus decrease the gut permeability (Camilleri et al., 2016).

Butyrate maintains the anaerobic environment in the colon by enhancing colonocyte oxygen consumption and stabilizing HIF, while its absence facilitates the buildup of potentially harmful bacteria and molecules, such as *Salmonella*, *E. coli*, and nitric oxide (NO), respectively (Parada Venegas et al., 2019). The reduced proportion of butyrate producers is also associated with a decreased count of methanogens, which disposes of the excess hydrogen (H_2_) produced in the form of CH_4_ during dietary fermentation, one of the possible reasons for the bloating experienced by IBS and IBD patients (Pozuelo et al., 2015; Chong et al., 2019). Studies have reported that among SCFAs, butyrate alone is responsible for gut motility, possibly *via* regulating serotonin, and can be used to increase propulsive gut movement, making it a suitable microbial therapeutic for patients with IBS (Vincent et al., 2018). An induced-colitis study in a murine model confirmed the decrease in butyrate-producing Clostridium clusters and reduced butyrate levels in the gut, which facilitated gut epithelial oxygenation and growth of *Salmonella enterica serovar Typhimurium* (S. *Typhimurium*), a known cause of foodborne gut inflammation and diarrhea (Rivera-Chávez et al., 2016; Anderson and Kendall, 2017; Litvak et al., 2019). Similarly, a reduced proportion of butyrate producers in the gut increases the expansion of aerobic Enterobacteriaceae, which is a common marker of gut dysbiosis (Matamouros et al., 2018; Parada Venegas et al., 2019). Studies have demonstrated a decreased count of butyrate-producing *Faecalibacterium* and *Roseburia* in the gut of ulcerative colitis patients (Sartor, 2011; Franzosa et al., 2019). On the other hand, the culture supernatant of *Faecalibacterium* was reportedly effective against IBD (Crohn’s disease) and colitis in murine models, and *Faecalibacterium* was found to secrete an anti-inflammatory peptide (MAM, m.wt. 15 KDa), which inhibits pro-inflammatory NF-κB signaling to arrest colitis (Quévrain et al., 2016). Additionally, *Faecalibacterium* inhibits colitis by producing anti-inflammatory shikimic and salicylic acids (Miquel et al., 2015). In another study, a combination of six different butyrate producers (*B. pullicaecorum* 25–3 T, *F. prausnitzii*, *Roseburia hominis*, *Roseburia inulinivorans*, *Anaerostipes caccae*, and *E. hallii*) reportedly enhanced butyrate production in IBD fecal microbiota by 5–10% and enhanced higher gut-barrier integrity, as examined in the Caco-2 cell line (Geirnaert et al., 2017). Similarly, patients with *Clostridium difficile* infection, which has a high mortality rate and increases the chances of acquiring hospital-acquired diarrhea, also exhibited a significant depletion in butyrate producers such as *Roseburia*, *Anaerostipes*, *Blautia*, and *Faecalibacterium*, along with lowered butyrate levels (Antharam et al., 2013). By contrast, in the case of mucositis, microbial butyrate enhances mucosal healing to accelerate the recovery of inflamed gut epithelium by stimulating the migration of gut epithelial cells (van Vliet et al., 2010).

By acting as a ligand, microbial butyrate participates in anti-inflammatory reactions to cease the inflammation and maintain gut homeostasis through the aryl hydrocarbon receptor (AhR) and various G-protein coupled receptors (GPCRs) such as GPR109a, GPR43, and GPR41 (Marinelli et al., 2019; Yip et al., 2021). AhR and GPCRs are transcription factors that control the transcriptional machinery of various immunoregulators following their activation. AhR exhibits the anti-inflammatory effect by enhancing anti-inflammatory IL-10 secreting B and Th2 cells, with a decline in pro-inflammatory Th1 and Th17 cells (Dong and Perdew, 2020; Abdulla et al., 2021). Among GPCRs, butyrate-activated GPR109a promotes differentiation of Treg cells and enhances anti-inflammatory IL-10 producing Th2 cells and plasma levels of IL-10, which in turn inhibits pro-inflammatory IL-17 (Akitsu and Iwakura, 2018; Martens et al., 2018). Upon butyrate activation, GPR43 reduces CD4 T-cell proliferation and limits the secretion of pro-inflammatory cytokines such as IL-17 and IL-22 (Kibbie et al., 2021). In addition, butyrogenic clostridia such as *Clostridium butyricum* limit IBD-associated inflammation by increasing Treg cell differentiation through microbial butyrate, which exerts its effects *via* transforming growth factor-β (TGF-β) (Ihara et al., 2017).

## Relevance of butyrate producers in CRC and tumorigenesis

Colorectal cancer (CRC) begins with a growth of the inner lining of the colon and rectum, which can later transform into cancerous polyps (Das et al., 2017; Salmerón et al., 2022). Evidence has shown that alterations in the gut microbiota are closely associated with CRC progression (Xie et al., 2020). Microbiome profiles of CRC patients exhibit a decrease in major butyrate-producing genera, including *Roseburia, Clostridiales, Faecalibacterium*, and members of the Lachnospiraceae family, and administration of butyrate-producing *Clostridium butyricum* was effective in decreasing the proliferation of cancerous cells and enhancing cancer cell apoptosis (Zou et al., 2018; Stoeva et al., 2021). Similarly, a lower abundance of *Eubacterium ventriosum* is a potential biomarker for CRC patients (Mukherjee et al., 2020), and its administration in CRC patients has been patented,^1^ indicating its significant therapeutic importance. Additionally, gut commensals such as *Butyricicoccus pullicaecorum*, *Butyrivibrio fibrisolvens*, *Ruminococcus bromii*, and members of the family Lachnospiraceae also produce sodium butyrate upon fermenting dietary fibers, which inhibits CRC cell proliferation by regulating immune cells such as natural killer cells and macrophages, and causes apoptosis (Xi et al., 2021).

**Figure 3:**
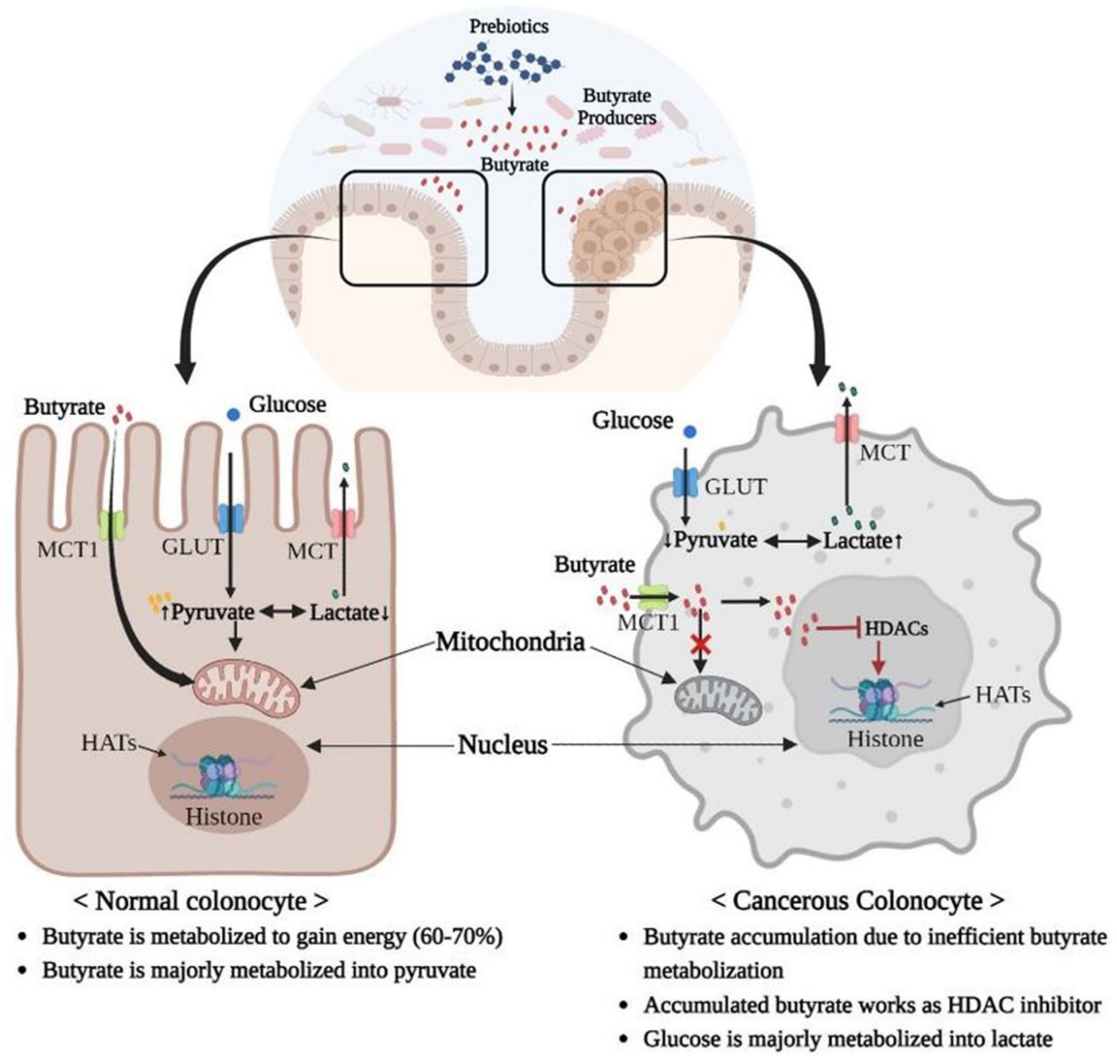
Warburg Effect: Inefficient butyrate metabolization by mitochondria of cancerous colonocytes leads to accumulation of butyrate, which in turn acts as an HDAC inhibitor and induces cancer. Additionally, majority of glucose is converted into lactate in cancerous colonocytes owing to their higher glycolysis rates, which is less energy efficient compared to phosphorylation of pyruvate in mitochondria *via* the TCA cycle. Therefore, cancerous colonocytes need higher glucose inflow and a higher rate of glycolysis to survive (MCT, Monocarboxylate Transporter; GLUT, Glucose Transporter; HDAC, Histone deacetylase, HAT; Histone acetyltransferase).

Luminal butyrate inhibits CRC mainly through HDAC inhibition by inactivating oncogenic pathways, such as mitogen-activated protein kinase (MAPK), Akt/ERK signaling, Wnt signaling pathway, and TGF-β signaling (Li et al., 2017; Geng et al., 2021). Butyrate-mediated inhibition of HDAC3 blocks the activation of Akt and ERK1/2, which are required for CRC cell migration and invasion (Li et al., 2017). Similarly, Wnt is a hydrophobic glycoprotein ligand that participates in various cellular processes, and aberration in Wnt signaling can cause CRC (Patel et al., 2019). An aberrant Wnt pathway can be suppressed by the butyrate-dependent activation of GPR109, as exhibited by *Clostridium butyricum*, but further investigation is required to confirm its direct or indirect role (Chen D. et al., 2020). Similarly, TGF-β is an immunosuppressive cytokine that regulates cell proliferation, differentiation, growth, and apoptosis, and any decrease in the inhibitory activity of TGF-β can lead to cancer, including CRC (Ku et al., 2007). Recent *in vivo* findings have reported significant expression of TGF-β after ingestion of dietary sodium butyrate, which can help combat CRC (Liu et al., 2014). Usually, cancer cells have a higher glucose demand and metabolic rate to support accelerated cell growth, which makes glycolysis inhibitors a promising anticancer drug candidate (Figure 3). Besides being an HDAC inhibitor, microbial butyrate differentially inhibits glucose transport, glycolysis, and DNA synthesis in cancerous colonocytes *via* inhibiting GLUT1 and glucose-6-phosphate dehydrogenase (G6PD) through the GPR109a-AKT pathway (Geng et al., 2021). GLUT1 is a glucose transporter, while G6PD is a key enzyme that produces ribose-5-phosphate for nucleotide synthesis (Geng et al., 2021). Microbial butyrate also inhibits CRC by increasing the 2-oxoglutarate level, which in turn downregulates proinflammatory cytokines such as IL-6, IL-22, IL1-β, and TNF-α (Wang et al., 2021). Furthermore, colonic butyrogenic microbes such as *Roseburia* and *Butyrivibrio* metabolize linoleic acid to produce the precursor of conjugated linoleic acid (CLA) (Devillard et al., 2007; Louis and Flint, 2009), which induces apoptosis and has been reported as an effective anti-carcinogenic molecule in various studies, including CRC (den Hartigh, 2019). *Roseburia* species, which are among the most active linoleic acid metabolizers, also produce vaccenic acid, which is known to be beneficial for the host (Devillard et al., 2007).

In contrast, some studies have reported an association between microbiota-derived butyrate and CRC upregulation (Okumura et al., 2021). This is a butyrate-paradox, wherein butyrate can act differently in normal and cancerous colonocytes. This is due to a metabolic shift of cancerous cells toward glycolysis, also called Warburg effect. In colonocyte mitochondria, butyrate is not metabolized to the same extent as in normal cells, and therefore, accumulates in the nucleus where it inhibits HDAC (Bultman and Jobin, 2014; Bultman, 2016; Hajjar et al., 2021; Figure 3). A similar paradox was observed in the microbial regulation of the PI3/Akt pathway, which is a major signaling cascade involved in the regulation of normal cellular activities, such as cell proliferation, growth, motility, and survival; however, its aberrant activation is associated with cancer (Luo et al., 2003; Prossomariti et al., 2020). Studies have reported that the PI3-Akt pathway is activated in 60–70% of CRC patients, and inhibitors of this pathway are considered therapeutic (Malinowsky et al., 2014). In the dysbiotic gut of CRC patients, the abundance of rare *Porphyromonas* species, such as *P. gingivalis* and *P. asaccharolytica*, may promote CRC *via* butyrate-mediated activation of the PI3/Akt pathway (Okumura et al., 2021).

## Relevance in gut-organ axis

Butyrate producers are associated with various gut-organ axes, such as the gut-brain, gut-lung, gut-liver, gut, kidney, and gut-heart axes (Ahlawat and Asha, 2021). In such complex relationships, butyrate producers act as microbial regulators and exert their effects through their metabolites. As in the gut-brain axis, microbiota-induced expression of AhR in gut neurons allows them to respond to the environment of the gut lumen while simultaneously connecting their functional output to the gut (Obata et al., 2020). As stated earlier, butyrate acts as a ligand for AhR, making butyrate producers a relevant community in the gut-brain axis. Studies have identified the antidepressant effects of the butyrate-producing genera *Butyricimonas* and *Coprococcus* and their depletion in depressed individuals (Yang et al., 2017; Valles-Colomer et al., 2019). Similarly, *Faecalibacterium* and *Coprococcus* are robustly associated with better mental health (Valles-Colomer et al., 2019). Metagenomic analysis of fecal samples from a Belgian cohort identified butyrate-producing *Alistipes* and *Roseburia* as potential producers of serotonin (Valles-Colomer et al., 2019), which is a neurotransmitter expressed abundantly in the gut where it regulates bowel movement, secretion (McLean et al., 2007), and glucose homeostasis (Singh et al., 2022). Studies also confirmed the gut-lung axis, as it’s been found that gut dysbiosis is closely related to the occurrence of asthma and pulmonary diseases. In infants reduced gut microbial diversity is reported to increases the risk of asthma and infectious respiratory diseases (Bisgaard et al., 2011; Abrahamsson et al., 2014). Specially, reduced abundance of butyrogenic *Faecalibacterium* in the gut is closely related with the increased risk of atopy and asthma (Dang and Marsland, 2019). In addition, during a viral infection such as influenza, through GPCR41 receptors, microbial butyrate enhances the Ly6C-monocytes in the lungs, which differentiate into alternatively activated macrophages (AAMs) that alleviate the immunopathological response in the lungs by limiting the neutrophil influx into the airways (Dang and Marsland, 2019).

The gut microbiome is also involved in the gut-liver axis because the liver receives approximately 70% blood supply from the gut, and even shows the presence of higher microbial liposaccharide (LPS) levels in the portal and hepatic circulation during chronic liver ailments (Compare et al., 2012). Microbial butyrate maintains the integrity of the gut barrier and inhibits the inflow of antigens (LPS). In murine studies, butyrate supplementation in the form of tributyrin was found to be effective in alleviating alcohol-induced liver injury (Cresci et al., 2017; Singhal et al., 2021). Alcohol-induced dysbiosis significantly reduces the members of Firmicutes and Lachnospiraceae with a lower abundance of butyrate-producing genera such as *Anaerostipes*, *Coprococcus*, and *Roseburia* (Singhal et al., 2021). A study based on a large human population (*n* = 1,148) also identified a significantly lower abundance of the genus *Faecalibacterium* in patients with non-alcoholic fatty liver disease (NAFLD) (Iino et al., 2019). Additionally, the butyrate-producing strain (MIYAIRI 588) of *Clostridium butyricum* reportedly suppresses oxidative stress and hepatic inflammatory indices in NAFLD (Endo et al., 2013).

Metabolites of protein fermentation, such as choline, phosphatidylcholine, and carnitine, are metabolized by the gut microbiota into trimethylamine, which is further converted into trimethylamine-N-oxide (TMAO) in the liver by hepatic flavin-containing monooxygenase (FMO) (Tong et al., 2022). TMAO is known to cause chronic kidney disease (CKD) and induces cardiovascular diseases such as atherosclerosis and coronary heart disease (Evenepoel et al., 2017). Although, a study also suggested that a low dose of TMAO might reduce cardiac dysfunction (Huc et al., 2018). Other than that, butyrate can lower the circulating cholesterol through reverse cholesterol transport by stimulating secretion of apoA-IV-containing lipoprotein (Chen W. et al., 2020). In addition, butyrate also enhances the secretion of glucagon-like peptide-1 (GLP-1) from the gut, which decreases blood pressure (Yadav et al., 2013). While, in CKD, the levels of uremic toxins such as indoxyl sulfate and p-cresyl sulfate are abnormally high, which can also lead to hypertension (Chen et al., 2019). Studies have reported decreased abundance of major butyrate producers such as *Roseburia*, *Faecalibacterium*, and *Coprococcus* in CKD patients (Jiang et al., 2017; Yang et al., 2018). In a murine study, CKD treatment with traditional medicine was found to be mediated by the butyrate-producing microbe Lachnospiraceae-NK4A136 *via* the gut-kidney axis (Tong et al., 2022). In addition to maintaining gut integrity to limit the level of uremic toxins, butyrate improves renal inflammation and dysfunction in patients with CKD.

## Impact of selective dietary interventions to enhance butyrate producers

Prebiotic administration positively affects butyrate producers, as they metabolize prebiotics into butyrate. Prebiotics are also beneficial in treating diarrhea and cholera, as prebiotic (e.g., resistant starch) administration accelerates recovery *via* microbial butyrate (Canani et al., 2011). Indigestible dietary fibers are commonly used as prebiotics, but other bioactive molecules, such as polyphenols, can also function as prebiotics to generate butyrate. Polyphenol intervention significantly increases the abundance of butyrate producers such as *Faecalibacterium* and members of the Ruminococcaceae family (Del Bo et al., 2021). Among other polyphenols, the impact of catechins, anthocyanins, and proanthocyanidins as prebiotics is more evident because they increase the abundance of *Roseburia* and *Faecalibacterium* spp. (Alves-Santos et al., 2020). Other phenolic compounds such as caffeic acid, chlorogenic acid, and rutin are also reported to increase microbial butyrate (Catalkaya et al., 2020). Additionally, the microbial accessibility of different prebiotics also varies among butyrate producers; therefore, the administration of different prebiotics can selectively enrich specific butyrate producers (Table 2). Other than prebiotics, synbiotic treatments can also be administered to promote butyrate production in the gut (Gurry, 2017). Synbiotics contain a combination of prebiotics and probiotics, and their synergistic effects are more prominent than those of prebiotics and probiotics used individually (Singh et al., 2021). Synbiotic treatment with *Bacillus subtilis* DSM 32315 and L-Alanyl-L-glutamine improved butyrate levels and enhanced the major butyrate producers such as *Faecalibacterium prausnitzii*, both *in vitro* and in humans (tom Dieck et al., 2022). Similarly, another study reported the prevalence of butyrate-producing *Eubacterium* and *Pseudobutyrivibrio* upon synbiotic administration of fiber-enriched yogurt (Jaagura et al., 2022).

**Table 2:** Impact of different fiber and bioactive metabolites on various gut butyrate producers.

Dietary substance	Monomer unit	Affected microbe	Model	Reference
Human milk oligosaccharides (HMOs)	β-d-galactose (Gal), β-d-glucose (Glc), β-d-N-acetyglucosamine (GlcNAc), α-l-fucose (Fuc), and the sialic acid α-d-N-acetylneuraminic acid (Sia)	*Roseburia*↑ *Eubacterium*↑	Human	Pichler et al. (2020)
Inulin	D-Fructose	*Faecalibacterium* ↑;	Human; Humanized mice	Healey et al. (2018), Van den Abbeele et al. (2011)
		*Roseburia intestinalis* ↑		
		*Eubacterium rectale* ↑		
		*Anaerostipes caccae* ↑		
Xylan	D-xylose	*Roseburia intestinalis* ↑	*In vitro*	Leth et al. (2018)
Fructooligosaccharide	D-fructose	*Faecalibacterium* ↑	Human	Tandon et al. (2019)
		*Ruminococcus* ↑		
		*Oscillospira* ↑		
Galacto-oligosaccharides	Galactose	*Anaerostipes caccae* ↑	Murine	Sato et al. (2008)
Polyphenols	Phenol	*Anaerobutyricum hallii*↑	Human	Del Bo et al. (2021)
		*Butyricicoccus* spp.↑		
		*Faecalibacterium prausnitzii*↑		
Pectin	Galacturonic acid	*Faecalibacterium*↑	*In vitro*	Bang et al. (2018), Chung et al. (2016)
		*Eubacterium eligens*		
Guar gum (Galactomannan polysaccharide)	Galactose and Mannose	Clostridium coccoides group↑	Human	Ohashi et al. (2015)
		*Roseburia/Eubacterium rectale* group↑		
		*Anaerobutyricum halli*↑		
		Butyrate-producing bacterium strain SS2/1↑		
Alginate	D-mannuronic acid and L-guluronic acid	*Bacteroides ovatus* ↑	*In vitro*	Li et al. (2016)
		*Bacteroides xylanisolvens* ↑		
Arabinoxylan	D-xylosyl	*Roseburia/Eubacterium rectale* group↑	Murine	Damen et al. (2011)
Stachyose	Galactose, Glucose, and Fructose	*Faecalibacterium*	*In vitro*	Zhao et al. (2021)
Lactulose	Galactose and Fructose	*Anaerostipes*	*In vitro*	Bothe et al. (2017)

## Strain and strategies for tomorrow

Butyrate-producing gut microbes are of significant therapeutic importance and are believed to be niche-specific next-generation probiotics. Multiple butyrate-producing probiotic strains of *Clostridium butyricum* (Stoeva et al., 2021) and *Butyricicoccus pullicaecorum* (Geirnaert et al., 2014; Boesmans et al., 2018) have been used as they exhibit good bile tolerance, viability, and metabolic activity (Table 3). Microbes of interest or butyrate producers can also be genetically manipulated to increase their butyrate-producing capacity. For example, heterologous genes required for butyrate production from acetyl-CoA can be introduced by inactivating the gene encoding the conversion of acetyl-CoA to acetate and the gene encoding the aldehyde/alcohol dehydrogenase for ethanol production or simply disrupting a CoA transferase gene, which may be an alternative route for acetate production (Ueki et al., 2014; Suo et al., 2018). Additionally, a co-culture strategy, that is an interactive microbial population of more than two microbes, can also be implemented to achieve higher levels of butyrate and increased abundance of butyrate producers in the gut. Co-culture of *F. prausnitzii* and *Bifidobacterium catenulatum* with fructooligosaccharides as an energy source resulted in a higher viable cell count and butyrate production (Kim et al., 2020). Moreover, butyrate producers of animal origin (ruminants), such as cellulose-degrading *Ruminococcus albus and R. flavefaciens* (Flint et al., 2008; Chassard et al., 2012), can also be considered to study their impact on human hosts.

**Table 3:** Butyrate producers that can be used as microbial therapeutic to maintain microbial homeostasis and gut health.

Microbes	Model	Reference
*Butyricicoccus pullicaecorum* 25-3^T^	Human	Boesmans et al. (2018)
*Faecalibacterium prausnitzii* A2-165	Murine	Martín et al. (2015)
*Eubacterium Hallii* DSM 3353	Human	Engels et al. (2016a)
*Eubacterium Hallii* DSM 17630	Human	Engels et al. (2016a)
*Eubacterium limosum* KIST612	Bio-fermenter	Litty and Müller (2021)
Co-culture of *Clostridium hylemonae DSM 15053*; or *Coprococcus comes ATCC 27758*; or *Roseburia hominis A2-183*; or *Eubacterium rectale ATCC 33656*; or *Eubacterium biforme DSM 3989* and *Clostridium ljungdahlii*	Dynamic metabolic modelling	Li and Henson (2021)
*Butyricicoccus pullicaecorum* 1.20; *Roseburia hominis* DSM 16839; *Roseburia inulinivorans* DSM 16841; *Anaerostipes caccae* DSM 14662; *Eubacterium hallii* DSM 3353	Fed batch fermenter and Caco-2 cell line	Geirnaert et al. (2017)
*Clostridium butyricum (CGMCC0313.1)*	Murine	Pan et al. (2019)
*Clostridium butyricum* (MIYAIRI 588)	Murine	Endo et al. (2013), Pan et al. (2019)
*Clostridium butyricum Prazmowski*	Murine	Wu et al. (2022)
*Ruminococcus albus*	Caco-2 cell line	Park et al. (2017)

## Conclusion

The present review critically examined all aspects of butyrate-producing gut microbial communities and their possible impact on host health to better understand their therapeutic significance. We considered the significance of butyrate producers and butyrate in the gut to understand their importance as microbial therapeutics. Although butyrate is an important metabolite, butyrate producers are much more important as they actively control the gut microbiome *via* various anti-microbial and anti-inflammatory molecules, and by synthesizing vitamin B. Butyrate-producing microbial communities inhibit cancer growth by secreting anti-carcinogenic substances and regulate tumorigenesis *via* butyrate. Butyrate producers are promising next-generation probiotics, and their counts in the gut can be regulated by dietary interventions to benefit the host. Moreover, butyrate producers can also be genetically manipulated to enhance butyrate synthesis, making them suitable microbial therapeutic agents. We also see the possibility of introducing new butyrate communities to the gut, which are alien to the human gut, to study their impact and to analyze any possible health effects. However, detailed studies are required to cease all safety concerns regarding the introduction of animal or soil origin butyrate producers in the human gut.

## Author contributions

VS conceptualized, analyzed, and wrote the draft. GL and HS participated in writing and project management. HK and EK supervised the manuscript. TU and J-HS supervised, reviewed, and approved the manuscript. All authors contributed to the article and approved the submitted version.

## Funding

This research was supported by Korea Basic Science Institute (National research Facilities and Equipment center) grant funded by the Ministry of Education (2021R1A6C101A416), and the Basic Science Research Program through the National Research Foundation of Korea (NRF) funded by the Ministry of Education (2016R1A6A1A03012862). This research was also supported by the project to train professional personnel in biological materials by the Ministry of Environment.

## Conflict of interest

The authors declare that the research was conducted in the absence of any commercial or financial relationships that could be construed as a potential conflict of interest.

## Publisher’s note

All claims expressed in this article are solely those of the authors and do not necessarily represent those of their affiliated organizations, or those of the publisher, the editors and the reviewers. Any product that may be evaluated in this article, or claim that may be made by its manufacturer, is not guaranteed or endorsed by the publisher.

## Footnotes

^1^ https://patents.google.com/patent/WO2016019506A1/en
